# Blunt trauma: An uncommon cause of common bile duct injury

**DOI:** 10.1016/j.tcr.2015.10.004

**Published:** 2015-11-06

**Authors:** Zachary Sanford, Kamran Abdolmaali, Dustin Robinson, David Denning

**Affiliations:** a Joan C. Edwards School of Medicine (JCESOM), Marshall University, Huntington, WV 25701; b Department of Surgery, JCESOM Marshall University, Huntington, WV 25701

**Keywords:** Blunt abdominal trauma, extrahepatic bile duct injury

## Abstract

Blunt force trauma to the extrahepatic biliary ductal system as a cause of avulsion is an uncommon injury associated with wide variability in prognosis. These cases are often difficult to identify, primarily as they are complicated by trauma patients exhibiting more immediate and obviously life-threatening injuries. This case demonstrates a 46 year-old-male involved in a head on motor vehicle collision, sustaining blunt force abdominal trauma resulting in partial transection of the common bile duct. Injury was discovered incidentally on exploratory laparotomy post endovascular repair of abdominal thoracic aortic rupture. Open cholescystectomy with intraoperative cholangiogram was performed, isolating extravasation from the common bile duct. A 16-French T-tube was placed in the common bile duct and two large #24 Jackson-Pratt tubes were placed in the vicinity. The procedure was well-tolerated and the patient was discharged with T-tube in place. Discharge was on postoperative day 28 with removal of tubes on postoperative day 54 and the patient was able to make a full recovery.

## Introduction

Disruption of the biliary tree secondary to blunt force trauma is a rare cause for extrahepatic bile duct injury [1], [2], [3]. Seldom occurring in isolation, this family of injuries is easily overshadowed by more overt surgical emergencies and can go undetected, potentially leading to adverse outcomes. A variety of imaging modalities have been used to varying degrees of success in identifying biliary tree disruption for stable patients but in emergent settings exploratory laparotomy remains the most efficacious means for identifying injury [4], [5]. This case presents an example of one such rare blunt force trauma shearing injury to the common bile duct in the setting of a motor vehicle collision.

## Case report

**Fig. 1:**
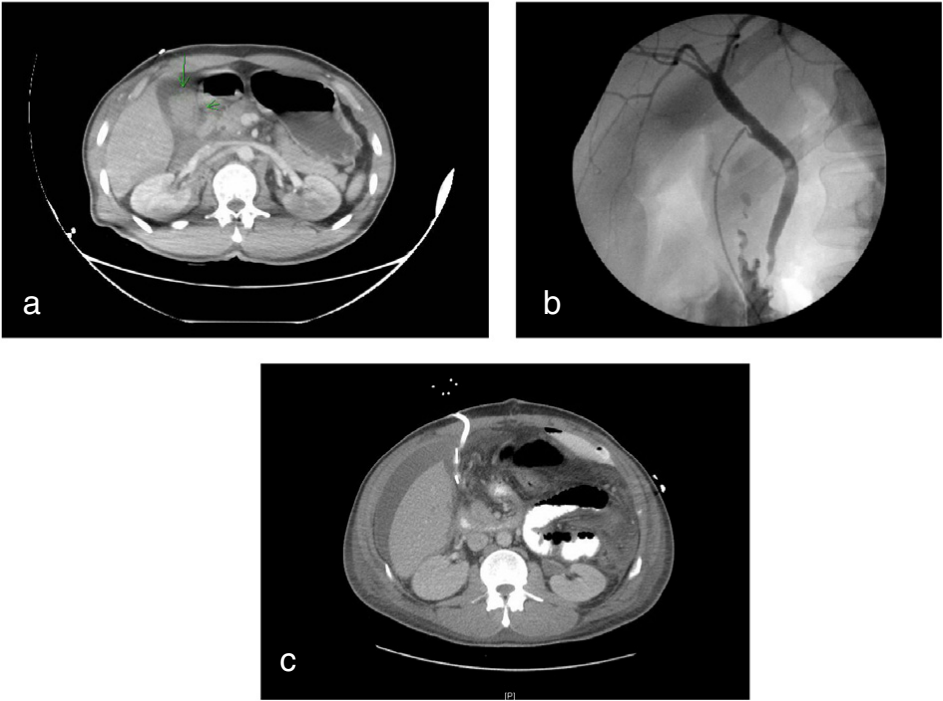
(a) Abdominal CT illustrating accumulation of blood in the gallbladder secondary to blunt force trauma sustained in motor vehicle collision (arrows); (b) Intraoperative cholangiogram with no evidence for common bile duct obstruction (radiolucent filling defect seen likely reflecting air bubbles in the duct); (c) Abdominal CT on postoperative day ten showing successful drainage of the operative site via T-tube.

A 46 year old male was an alcohol-intoxicated restrained passenger in a head-on motor vehicle collision, suffering blunt force abdominal trauma. At the collision site EMS recorded a Glasgow Coma Score of 15 with stable vital signs. He was transferred to Cabell Huntington Hospital where he was intubated and found to be tachycardic and profoundly academic to an arterial pH of 7.19 with accompanying base deficit of -11. Hemoglobin was 12.9, hematocrit 37.1, PT 11.5 sec, and APTT 23.5 sec. CT showed acute thoracic aortic rupture with a large volume of blood inferiorly along the aorta, right middle and lower lobe lung contusions, and a 4 cm laceration along the inferior lobe of the liver with blood in the renal hilum suggesting right renovascular injury and free fluid within the pelvis (Fig. 1a). He was transferred to Saint Mary’s Medical Center for emergent management of aortic rupture.

**Fig. 2:**
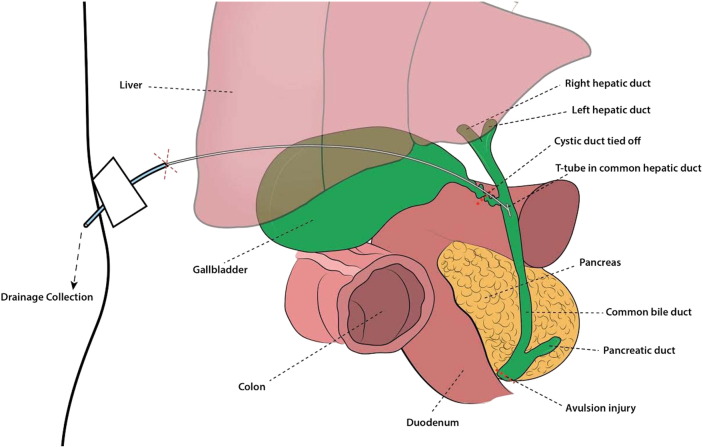
Representation of blunt force abdominal trauma sustained in a head on motor vehicle collision resulting in partial transection of the common bile duct. Injury was discovered incidentally during exploratory laparotomy status post repair of thoracic aortic rupture.

Emergent surgical management of the ruptured thoracic aorta was completed by the cardiothoracic team. Upon completion, initiation of exploratory laparotomy was begun, noting blood in the peritoneum and bile staining in the right upper quadrant associated with the transverse and hepatic flexures of the colon. The gallbladder was visibly distended, firm to palpation, and discolored but showed no signs of rupture. Subcapsular hematoma was appreciated along the right posterior lobe of the liver but showed no signs of actively expanding or gross laceration. Open cholescystectomy with intraoperative cholangiogram was performed on a thick, distended and grossly-discolored gallbladder consistent with hemorrhage into the gallbladder lumen (Fig. 1b). Conray contrast was used to demonstrate a completely intact biliary tree with extravasation noted from the common bile duct. Duodenal injury was ruled out by passing methylene blue through the orogastric tube with no extravasation appreciated and colon was assessed via direct visualization by Kocher maneuver and mobilization of the hepatic flexure. Due to the duration of the thoracic aorta repair and the exploratory laparotomy, the decision was made to place a 16-French T-tube in the common bile duct in addition to two large #24 Jackson-Pratt tubes in the vicinity of the bile duct (Fig. 2). The patient was maintained on ventilator in the neurotrauma intensive care unit. In the postoperative period the T-tube drained golden brown bile and was well-tolerated.

Urine collection via Foley catheter showed blood-tinged urine that persisted until POD 10 but then spontaneously resolved. During this time on POD 6 the patient underwent gastrostomy with placement of percutaneous gastrostomy and tracheostomy tubes. Due to the PEG becoming disloged on POD 8 the patient underwent exploratory laparotomy and gastrostomy with jejunostomy tube placement and removal of foreign body. Evaluation on postoperative day 10 showed successful drainage of the operative site via T-tube (Fig. 1c). The patient continued to improve and was discharged on POD 28 with T-tube and J tubes in place.

Recovery was complicated during the outpatient course as frank blood began appearing in the patient’s urine on POD 39 and the patient began complaining of pain around tube sites. CT urogram showed no residual renal injury and was presumed to be due to residual posttraumatic blood accumulation. Of note, a small amount of fluid was appreciated in the anterior right middle and upper abdomen along the inferior right lobe of the liver not seen previously but consistent with postoperative seroma. A urinary tract infection was diagnosed and managed medically. T tube cholangiogram revealed no abnormalities and the the T-tube was removed along with J-tube on POD 54. The remainder of the patient’s recovery period was uneventful with regards to hepatobiliary procedures, however frank blood in the urine persisted for an additional 6 months due complications of subclinical traumatic kidney injury sustained during the motor vehicle collision.

## Discussion

Injuries of this nature are complex and, due to their infrequency, often evade detection by trauma physicians. Incidence of extrahepatic biliary duct injury in the setting of blunt force trauma has been reported to be as low as 1 in 10,500 consecutive trauma cases [6], with the first case being reported in 1799 [5]. Proper diagnosis requires a high index of suspicion and without an agreed upon universal protocol for management outcomes are widely variable [7], [8], [9].

Ductal injury often localizes at one of three anatomic sites: the origin of the left hepatic duct, the bifurcation of the hepatic ducts, or the panceaticoduodenal junction [10], [11]. We report a case of common bile duct injury at the third of these anatomic sites that was identified by a combination of CT and exploratory laparotomy. Although imaging modalities such as CT and ultrasound can result in significant false negatives, more sensitive modalities such as cholangiotransparietohepatography (CTPH), endoscopic retrograde cholangiography (ERCP), and MRI are more appropriate in non-emergent settings [8]. Other modalities, such as percutaneous aspiration and isotope scintigraphy such as with hepatobiliary iminodiacetic acid (HIDA) are also options for nonemergent evaluation however diagnostic value is limited due to their inability to specifically localize site of injury [10], [12].

In the absence of hemodynamic instability assessed by exploratory laparotomy, injury can be overlooked. The presence of jaundice after blunt force trauma should raise concerns for biliary tree injury and, while nonspecific, generalized symptoms such as worsening abdominal discomfort, nausea, vomiting, low-grade fever and persistent ileus can help raise suspicion [13]. Due to the delay in onset of these symptoms, morbidity rates in these cases can approach 40%, including bleeding, infection, and compartment syndrome [9], [10]. Even when managed urgently, surgical complications can result in postoperative anastomotic leakage, recurrent cholangitis with or without stricture, biliary cirrhosis, and portal hypertension [10], [14].

Due to the infrequency of this injury in abdominal blunt force trauma cases, clinicians should be weary to remember the extrahepatic biliary ductal system can perforate or even completely transect even without other obvious thoracoabdominal injury, potentially evading detection for some time and possibly complicating course of recovery. Physicians should maintain a high index of suspicion with deteriorating posttraumatic patients exhibiting biliary obstruction symptoms in the nonemergent patient and should always look for bile staining or signs of biliary perforation when surgically repairing emergent cases.

## Conflicts of interest

No members of the research or clinical team have any stated or potential conflicts of interest.
